# A Designed Broadband Absorber Based on ENZ Mode Incorporating Plasmonic Metasurfaces

**DOI:** 10.3390/mi10100673

**Published:** 2019-10-04

**Authors:** Phuc Toan Dang, Khai Q. Le, Ji-Hoon Lee, Truong Khang Nguyen

**Affiliations:** 1 Division of Electronics Engineering, Chonbuk National University, Jeonju 54896, Korea; dangphuctoan@jbnu.ac.kr; 2 Division of Computational Physics, Institute for Computational Science, Ton Duc Thang University, Ho Chi Minh City 700000, Vietnam; lequangkhai@tdtu.edu.vn; 3 Faculty of Electrical and Electronics Engineering, Ton Duc Thang University, Ho Chi Minh City 700000, Vietnam

**Keywords:** epsilon-near-zero, wideband absorber, plasmon mode, Brewster mode

## Abstract

In this paper, we present a numerical study of a metamaterial absorber that provides polarization-insensitive absorption over a broad bandwidth of operation over the mid-infrared region. The absorber consists of a periodically patterned metal-dielectric-metal structure integrated with an epsilon-near-zero (ENZ) nanolayer into the insulating dielectric gap region. Such an anomalous broadband absorber is achieved thanks to a couple of resonant modes including plasmon and ENZ modes that are excited under mid-IR light illumination. By adding a 0.06-μm-thick ENZ layer between the patterned gold rectangular grating and the SiO_2_ dielectric layer, the absorber captures >95% light over a 1.5 µm bandwidth centered at a near-8-μm wavelength over a wide range of oblique incidence under transverse-magnetic and -electric polarizations. The designed ENZ-based wideband absorber has potential for many practical applications, including sensing, imaging and solar energy harvesting over a wide frequency regime.

## 1. Introduction

Nowadays, perfect light absorption has attracted much attention for the majority of the modern optoelectronic devices working either in the infrared regime or in the visible spectrum. Among these, perfect wideband absorption has attracted great attention due to its high applicability in practical applications such as surface-enhanced sensing and imaging sensors. Especially, in solar cell applications, high-performance photodetectors all rely on absorption of light. The absorption spectrum can be adjusted in terms of absorption strength and bandwidth and can expand the range of many practical device applications. So, a number of techniques have been proposed over the past years to design the absorption of materials, examples of which include metamaterials, ultrathin semiconductors, coherent absorption and plasmonic perfect absorber [1,2,3,4,5,6,7,8,9,10,11,12]. 

Up to now, most of the research related to wideband absorption has mainly been based on plasmonic resonances of nanostructures having delicate structural patterns [13,14], or some studies based on epsilon-near-zero (ENZ) metamaterial structures which can also achieve wideband perfect absorption [15,16]. The complete light absorption in thin films allows perfect wideband carrier collection thanks to the mutual interaction between plasmonic nanostructures and semiconductors absorbers, as shown in previous studies [17,18,19]. In addition, the omnidirectional broadband absorption can be obtained by nonresonant plasmonic Brewster effects [20]. 

Recently, combinations of the gap plasmonic mode and the ENZ mode have been emerging as good candidates to optimize broadband absorption [21]. However, the coupling regime of the mode is still a weak and narrow bandwidth. In this work, we present a study of ENZ-enhanced wideband absorption near the ENZ wavelength in the mid-infrared region when an ENZ material is integrated into the gap region of a metal-insulator-metal plasmonic structure. Our study is based on the combination of the gap plasmon mode, ENZ mode, and plasmonic Brewster effects. A mode called "epsilon-near-zero" (ENZ) has been observed on subwavelength film thicknesses. The ENZ mode is also known as a Berreman mode which can appear only as small film ENZ thicknesses [22]. The role of ENZ layers and losses in such materials has been also discussed in previous studies [23,24,25,26]. 

In detail, the requirement for ENZ mode operation is that their thickness must be on the order of or less than the wavelength where the real part of the dielectric constant becomes zero [27]. ENZ modes have shown very large densities of states which make them attractive for enhancing light–matter interactions. Moreover, by varying the doping concentration (i.e., depending on the doping level) or other growth conditions, the ENZ wavelength can be controlled in a definite spectral range in the near- and mid-infrared bands [28,29]. One such ENZ material is InAsSb [27], with an ENZ wavelength that can be generated at around 8 μm. At this wavelength, the electric field in plasmonic subwavelength thin films becomes very strong, and this can lead to extremely large light absorption in the film [10]. Several studies have demonstrated that thin films made of metal, doped semiconductors, or polar materials can support surface plasmon polaritons (SPPs) [30,31]. The gap plasmon resonance localized in the dielectric gap region between two metal layers, which has a strong electric field oriented in the out-of-plane direction [32], can be incorporated efficiently to the ENZ mode of the ENZ layer. 

The plasmonic Brewster effects are based on an inherently non-resonant mechanism. At a specific angle, the Brewster angle, light absorption can be achieved over broad bandwidth thanks to the impedance-matching mechanism at the entrance and exit metal surfaces with minimizing reflections through the corrugated metal screen [20]. In this study, we have optimized ENZ mode, plasmon mode, and Brewster effect. These resonances have been controlled to be efficiently coupled together in close proximity. As a result, high and wideband absorption is achieved simultaneously in the wavelength range of interest.

## 2. Absorber Structure and Simulation Details

The absorber device structure was designed on a 2-μm supporting silicon substrate. As for the silicon dioxide dielectric spacer layer, there are two considerations when calculating thickness. The first consideration is that the spacer layer thickness affects the absorption intensity of the gap plasmon resonance, as has been seen in many previous studies on metal-insulator-metal structures without an integrated ENZ layer [33]. The second consideration is that too thick of a spacer layer makes the coupling of the gap plasmon mode to the ENZ mode weaker because of the spatial overlap of these modes decreasing gradually with the increasing thickness of the spacer layer. Contrariwise, if the spacer layer thickness is too thin, the coupling regime is made robust and can split the two resonances in the hybrid resonance modes [34]. Choosing the exact thickness of the spacer layer depends on the thickness of the ENZ layer. 

**Figure 1:**
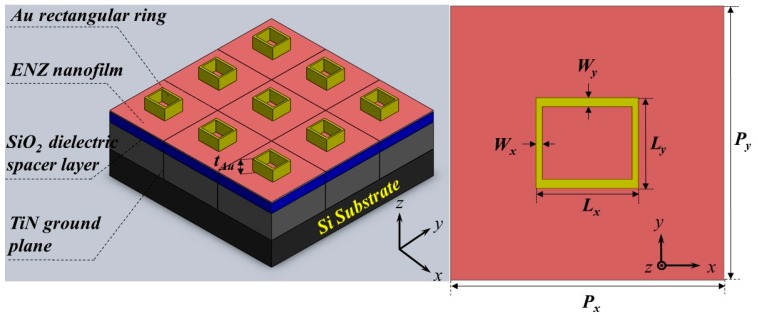
Sketch and top view of the epsilon-near-zero (ENZ)-based absorber with a top rectangular grating.

Figure 1 illustrates the proposed absorber structure with the following specific parameters. The exact thickness of the dielectric layer was 0.75 μm to separate the two metal layers. The repeat period *P* was 4.55 μm in both x and y directions. A metallic TiN film worked as a ground plane. The gold gratings were placed on the top of the ENZ nanofilm, which has the resonance wavelength of 8 μm. For the device, the absorption band can be broadened significantly in the mid-infrared region around the ENZ resonance wavelength. The thickness of the ENZ layer was 0.06 μm. On the top layers, the height, the lengths of the rectangular grating are designated as *t*_Au_, *L_x_* and *L_y_* in the *x* and *y* directions with values of 0.9, 1.59 and 1.5 μm, respectively. 

Similarly, the thicknesses of the rectangular grating’s rim *W_x_* and *W_y_* were 0.09 μm and 0.1 μm, respectively. Optical properties of TiN, SiO_2_, Si layers were extracted from the material library of the simulation software and verified with experimental data [27], while the gold electric permittivities were taken from Palik’s Handbook of Optical Constants [35]. Dispersive properties of the constituent materials of the device absorber were taken into the simulations. 

**Figure 2:**
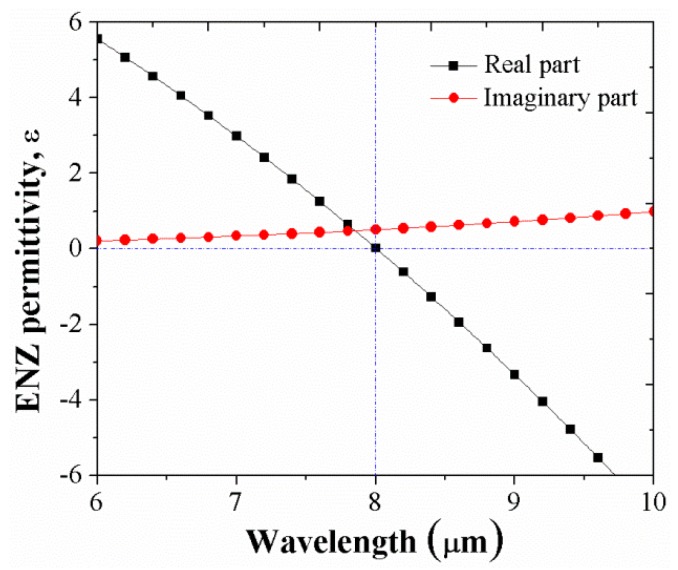
The dielectric constant of the ENZ material.

ENZ materials are known to possess some unusual properties when a class of the materials which have their real part of permittivity crossing zero at a certain wavelength (i.e., ENZ wavelength) [27]. Figure 2 shows the resultant spectral dependence of the real and imaginary parts of the ENZ permittivity (ε). In order to demonstrate ENZ-enhanced wideband absorption, we show the coupling of the gap plasmon resonance mode which localized in the gap SiO_2_ layer with a strong electric field region and ENZ mode which was excited into the gap region of metal-insulator-metal interfaces. In addition, the effects of angles of incident and other parameters in the structure were also considered.

## 3. Results

A significant factor in getting broadband absorption is the coupling between the gap plasmon mode and the ENZ mode must be robust, but still be weak coupling. Therefore, the simultaneous combination of these two resonances and the Brewster mode has been investigated in repelling each other to generate wideband absorption. Moreover, the wideband absorption also has an incident angle and therefore it is more suitable for many device applications that require multiple-direction light absorption and energy conversion. We utilized the commercial CST Microwave Studio (CST MWS; Computer Simulation Technology AG., Darmstadt, Germany) software based on a finite element method (FEM) [36] to simulate the device with appropriate boundary conditions. In the device considered here where an optical TiN thick metallic ground plane is used (i.e., transmission is equal to zero), the simple expression of A = 1 − R will show the relationship between the absorption A and reflectivity R. This section will provide a concise and precise description of the simulated results, their mechanism as well as the conclusions that can be drawn. 

**Figure 3:**
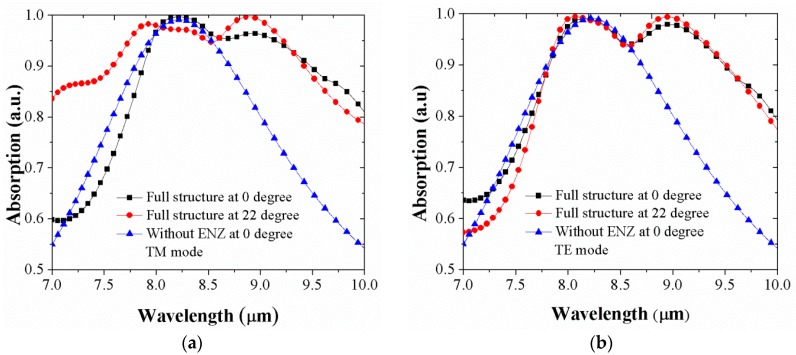

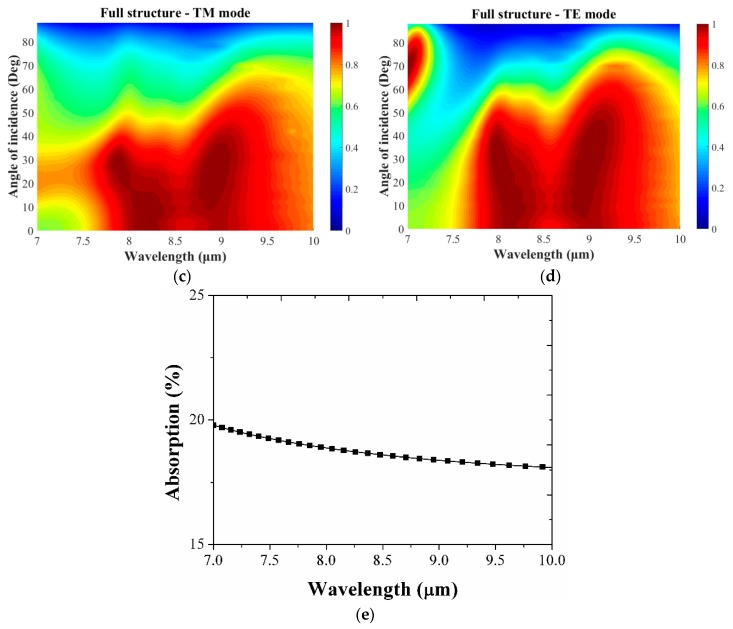
Simulated characteristics of the absorber structures for different incident angles. (**a**,**c**) transverse-magnetic (TM) mode; and (**b**,**d**) transverse-electric (TE) mode; and (**e**) the percentage of absorption only in the metal as a function of wavelength.

The absorption spectrum of the integrated ENZ wideband absorber is also shown at the normal incident (black square line) and at the Brewster angle (red circle line) with a wideband absorption of approximately 95% over wavelength ranges of 1.26 μm (from 7.94 μm to 9.2 μm) and 1.46 μm (from 7.77 μm to 9.23 μm), respectively, shown in Figure 3a for transverse-magnetic (TM) polarization. Along with that comparison, in order to reobtain the perfect absorption spectrum in the device without the ENZ layer, herein called a “ENZ-removed” perfect absorber, it was necessary to change the device parameters to a 0.55-μm spacer layer, a 0.4-μm grating thickness, lengths of the grating (*L_x_* and *L_y_*) which have same value of 1.31 μm and thicknesses of grating’s rim (*W_x_* and *W_y_*) being 0.11 μm in the x and y directions; all other parameters remained the same. The calculated bandwidth for 95% absorption is 0.58 μm (from 7.94 μm to 8.52 μm) for the “ENZ-removed” perfect absorber (blue triangle line) also shown in Figure 3a for TM mode. A similar comparison is shown in Figure 3b for transverse-electric (TE) mode with various spectral absorption ranges. The angles of incidence play an important role in the optical response of nanostructured surfaces. Degraded performance stems from large oblique angles. To understand more about the performance of the optimized integrated wideband ENZ absorber, we calculated the absorption as a function of the incident angle over a range of 0–90° for both TE and TM modes for the structure. The full structure or the optimized integrated wideband ENZ absorber structure exhibited angular stability up 24° with wideband absorption intensity of over 95% as shown in Figure 3c for TM mode. The absorption gradually decreased with increase in incidence angle. Similarly, the wideband absorption and absorption intensity were reduced when incident angles were over 22° for TE mode as shown Figure 3d. For both TE and TM modes, the absorption intensity on the shorter wavelength side of the absorption bandwidth began to decrease as the incident angle increased above these bandwidths, while the long-wavelength side of the absorption band remained until below 70°. Figure 3e shows the percentage of absorption in the metal layers of the structure where the absorption loss was around 20% in the wavelength range of interest. It can be seen that the absorption bandwidth in the TM mode was wider than that of the TE mode which is due to the temporal and spatial interference between the optical cavity in the SiO_2_ spacer and the grating plasmonic modes modifying the angular response. This can be seen in Figure 3a,b where the absorption bandwidths in TM and TE modes are almost same at the normal incident. Though the plasmon lifetime also affects such an absorption bandwidth [5,6], this effect was minor in this proposed design.

**Figure 4:**
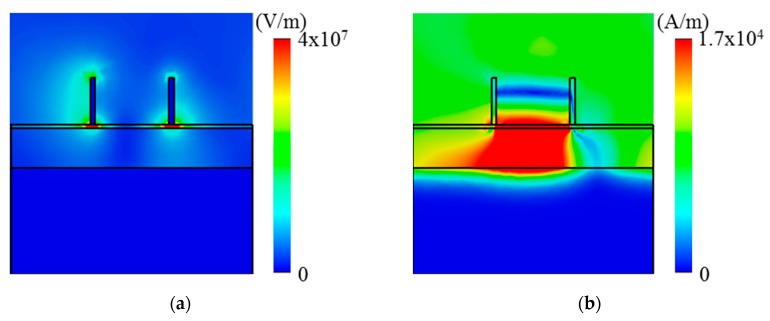
(**a**) E-field magnitude distribution (in units of V/m) indicating ENZ mode at 7.9 μm wavelength and (**b**) H-field magnitude distribution (in units of A/m) indicating the gap plasmon mode at 8.9 μm wavelength within the perfect absorption band.

The absorption mechanism of the structure can be explained by combination of plasmonic effect, ENZ mode and Brewster mode. These effects cause the strong electromagnetic resonances at the absorption wavelengths. As shown in Figure 2, when the dielectric constant ε becomes zero (i.e., ENZ) at near 8 μm we could still achieve reasonably high field enhancement at the ENZ wavelength. As an effective explanation for the mechanisms of the observed absorption of three peaks of light, Figure 4 shows the electromagnetic field distributions at the specific resonant wavelengths. At 7.9 μm, the first absorption peak, we investigated the distribution of the magnitude of the electric field. As a result, the electric field was excited and confined strongly into the ENZ layer, as shown in Figure 4a. This behavior indicates that the excitation of the ENZ mode has occurred. Figure 4b shows the magnetic field magnitude distribution of the third peak at 8.9 μm. It can be found that the magnetic field enhancement is mainly located in the dielectric gap between the gold grating and the TiN metallic film, indicating that the gap plasmon resonance mode is strongly excited. This effect can be understood to be simply due to the patterned gold grating and the ENZ layer generating electric fields and currents when the structure has the presence of an electromagnetic wave. The high charge concentration at the edges of the gold grating results in the strong enhancement of the field around the edges of the metal structure. In the opposite direction, the conductive TiN ground plane generated the currents and electric fields thanks to the near-field coupling. Then, the antiparallel currents generated magnetic fields in the SiO_2_ layer. 

In the structure, we set up a periodic structure with boundary conditions and the incident waves excited along the z-axis by using Floquet ports at the top and bottom of the structure. When the light propagates as a plane wave on this structure, light will be reflected or absorbed depending on the mismatch of impedance in this structure to impedance in free space. By using numerical analysis, the effective refractive index *n* and the effective impedance *z* can be extracted from the relationship with S-parameters expressed in Equations (1)–(4), where $S_{11}$ and $S_{21}$ are the S-parameter results from the two-port model of the aforementioned structure, $k_{0}$ is wave number and *m* is an integer [37].

(1) $$S_{11}=\frac{\Gamma \left(1-e^{i4nk_{0}d}\right)}{1-\Gamma^{2}e^{i4nk_{0}d}}$$

(2) $$S_{21}=\frac{\left(1-\Gamma^{2}\right)e^{i4nk_{0}d}}{1-\Gamma^{2}e^{i4nk_{0}d}}$$

(3) $$z=\pm \sqrt{\frac{{\left(1+S_{11}\right)}^{2}-S_{21}{}^{2}}{{\left(1-S_{11}\right)}^{2}-S_{21}{}^{2}}}$$

(4) n=12k0d{[Im(ln(ej2nk0d))]+2mπ−i [Re(ln(ej2nk0d))]}

Similar to the explanation of the mechanism of metamaterial absorber structures in previous studies, the mechanism of the behavior of this device is also explained through impedance matching mechanism [38,39]. In this mechanism, the absorber structure plays a role as a thin slab which made from a homogeneous structure. The mechanism of absorption in the structure is due to the coupling of resonance modes to Brewster mode to generate the strong electromagnetic resonances at the absorption peaks as explained in the above section. Also, we utilized the effective medium theory to explain the absorption mechanism. We calculated the effective parameters of the structure through reflection and transmission coefficients of the structure. The relationship of the thickness of a homogeneous slab (*d*), free space vector (*k*_0_), S-parameters and the effective parameters of the input impedance (*z*) and the refractive index (*n*) as expressed in Equation (3). However, this structure is backed by a metallic layer, therefore, Equation (3) will become:

(5) $$z=\pm \sqrt{\frac{{\left(1+S_{11}\right)}^{2}}{{\left(1-S_{11}\right)}^{2}}}=\left|\frac{1+S_{11}}{1-S_{11}}\right|$$

**Figure 5:**
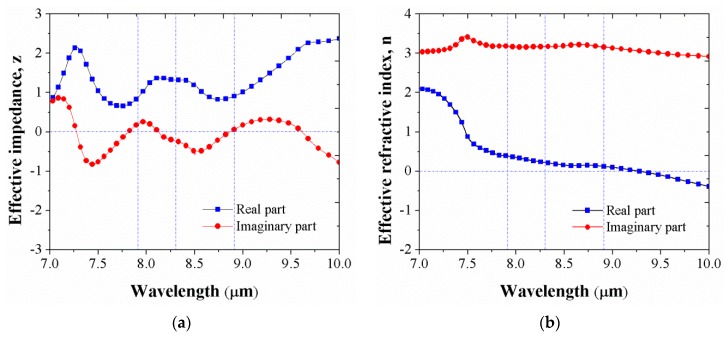
(**a**) Effective impedance and (**b**) effective refractive index for ENZ-based absorber model.

Figure 5a shows the effective impedance at resonance wavelengths. Figure 5a indicates that the absorption is maximum when the real part of the impedance of the structure is equal to free space (i.e., equal to 1) and the imaginary part has a value of zero. Otherwise, the absorption will be reduced due to the impedance mismatch between the structure and free space, which results in reflection. Meanwhile, the effective refractive index is given Equation (4). In Figure 5b, at the perfect absorption wavelength of 8.9 μm, the effective refractive index exhibits a large imaginary part, $n^{\prime \prime }>>n^{\prime }$. $n=n^{\prime }+in^{\prime \prime }=0.12\;+\;3.16i\;$ has the large imaginary part (i.e., $n^{\prime \prime }=3.16,n^{\prime }=0.12$) with a high ratio contrasting the real part and the imaginary part of up to 26.3, so that the incident electromagnetic wave enters the device without any reflectivity and then rapidly decays to zero inside the structure.

When light propagates between two media of differing refractive indexes, generally some of it is reflected at the boundary. However, light with one particular polarization cannot be reflected at one particular angle of incidence. This angle of incidence is Brewster’s angle $\theta_{B}$ which can be determined by:

(6) $$\theta_{B}=\arctan\left(\frac{n_{2}}{n_{1}}\right)$$

This equation is called Brewster’s law in which *n*_1_ is the refractive index of the incident medium (usually air) and *n*_2_ is the effective index of the medium in the structure. In this work, we investigated a light wave passing from air (*n*_1_ = 1.00) to the effective medium (*n*_2_ = 0.41) at a wavelength of 7.9 μm; the Brewster angle, $\theta_{B}$, was calculated to be 22°.

**Figure 6:**
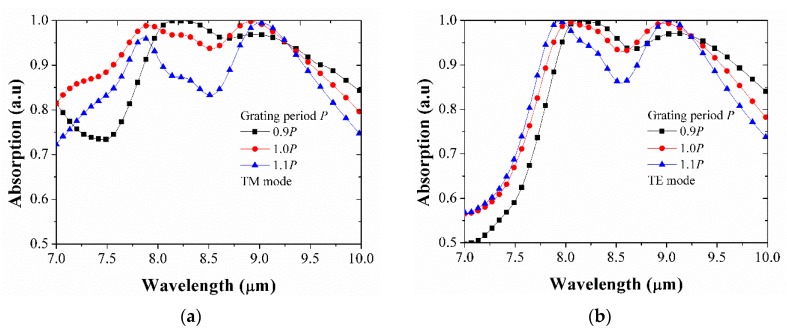
Investigation of the effect of periodicity *P* for absorption light in the structure. (**a**) for TM mode, (**b**) for TE mode.

We have also investigated the effects of periodicity (*P*) on the absorption light in the structure by increasing and decreasing it by 10% compared to the optimized value of P for both TM mode and TE mode. Figure 6 shows the absorption characteristics for various periodicities. In general, the absorption peaks shifted, and absorption intensity was also affected, especially at the absorption peak around 8 μm, as *P* varied, which was observed both in TM and TE modes. The optimized *P* was selected regarding to the highest absorption and widest bandwidth.

## 4. The Absorptivity as a Function of Polarization angle

**Figure 7:**
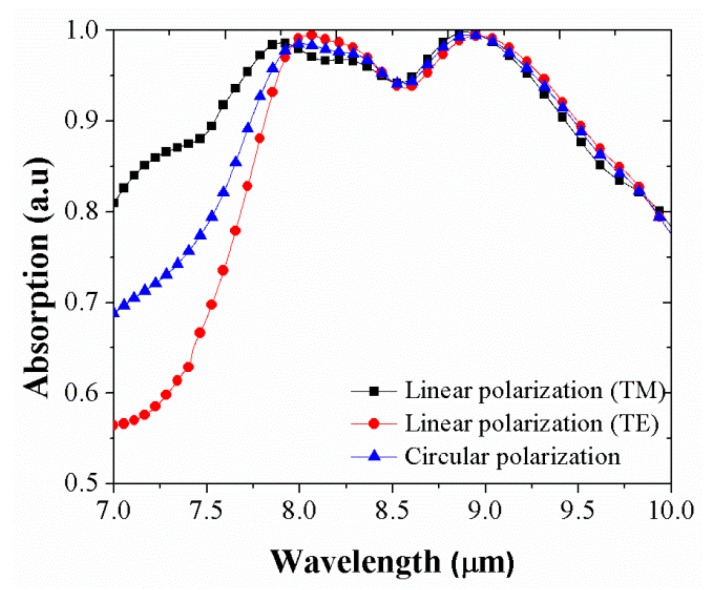
Comparison of TM mode, circular polarization and TE mode for simulated absorption curves from devices of integrated ENZ perfect absorber at Brewster angle.

The insensitivity of polarization is an essential factor in practical applications. In this paper, the polarization independence of the proposed absorber was investigated for polarization angle. Figure 6 shows plots of the absorption spectra of the proposed light absorber to demonstrate the absorption spectra for the different polarizations. Optimization of the integrated ENZ wideband absorber can be observed at the Brewster angle for the TM and TE modes and circular polarizations; such an absorber shows the spectral band with 95% approximate absorption over a 1.46 μm spectral range (from 7.77 μm to 9.23 μm) for TM mode (black square line) and over a 1.4 μm wideband absorption range (from 7.78 μm to 9.27 μm) for TE mode (red circle line). As a comparison, the absorption spectrum of the integrated ENZ wideband absorber is also shown at the Brewster angle (blue triangle line) for circular polarization with a wideband absorption of approximate 95% over a wavelength range of 1.26 μm (from 7.94 μm to 9.2 μm). It can be found from Figure 7 that for both TE and TM polarized waves and circular polarization, the absorption curves are quite similar, which reveals that the proposed absorber is polarization-insensitive. 

**Figure 8:**
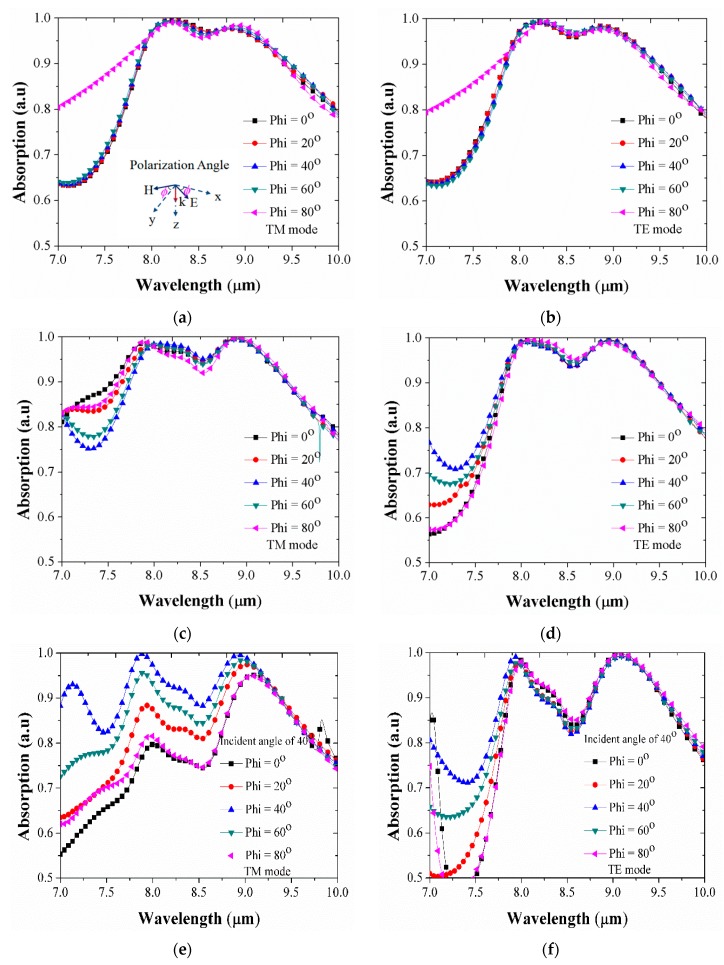
Absorption curves as a function of polarization angle (phi) under (**a**,**b**) normal incidence; (**c**,**d**) Brewster angle; (**e**,**f**) and 40° incidence.

Next, we also investigated the polarization independence of the proposed absorber for polarization angle. Figure 8 illustrates the absorption spectra with various polarization angles for the both TM and TE modes at normal incident, Brewster angle, and 40° incident. The absorption curves are similar, which indicates that the enhanced absorption is independent of the light polarization, at least up to Brewster angle. However, beyond this angle, TE mode can keep its polarization-insensitivity but the TM mode cannot. 

## 5. Conclusions

We have proposed an absorber based on a combination of ENZ mode and the gap plasmon mode and Brewster effect. The simulated results reveal that this device has a wide range absorption bandwidth of mid-infrared radiation (IR) wavelengths for polarized waves at normal incident and Brewster angle. Furthermore, the ENZ-based absorber is polarization insensitive and keeps high absorption in a broad wavelength range at oblique incidence for TM and TE polarization wave. The proposed design is expected to work at other different wavelength ranges based on the studied mechanisms. The ENZ-based absorber will be a potential candidate for many applications, such as detection, sensing, imaging and defense applications. Also, our device can help improve light-harvesting efficiency with enhanced absorption both in terms of intensity and broadband.
